# Predictors of Traditional Medical Practices in Illness Behavior in Northwestern Ethiopia: An Integrated Model of Behavioral Prediction Based Logistic Regression Analysis

**DOI:** 10.1155/2017/4186064

**Published:** 2017-10-18

**Authors:** Abenezer Yared

**Affiliations:** Department of Sociology, College of Social Sciences and Humanities, Ambo University, P.O. Box 19, Ambo, Oromia, Ethiopia

## Abstract

This study aimed at investigating traditional medical beliefs and practices in illness behavior as well as predictors of the practices in Gondar city, northwestern Ethiopia, by using the integrated model of behavioral prediction. A cross-sectional quantitative survey was conducted to collect data through interviewer administered structured questionnaires from 496 individuals selected by probability proportional to size sampling technique. Unadjusted bivariate and adjusted multivariate logistic regression analyses were performed, and the results indicated that sociocultural predictors of normative response and attitude as well as psychosocial individual difference variables of traditional understanding of illness causation and perceived efficacy had statistically significant associations with traditional medical practices. Due to the influence of these factors, majority of the study population (85%) thus relied on both herbal and spiritual varieties of traditional medicine to respond to their perceived illnesses, supporting the conclusion that characterized the illness behavior of the people as mainly involving traditional medical practices. The results implied two-way medicine needs to be developed with ongoing research, and health educations must take the traditional customs into consideration, for integrating interventions in the health care system in ways that the general public accepts yielding a better health outcome.

## 1. Introduction

Illness behavior, broadly speaking, refers to any behavior undertaken by an individual who feels ill to relieve that experience or to better define the meaning of the illness experience. The study of illness behavior, therefore, involves investigating the way people define and interpret bodily indications and use informal and formal sources of care [1], and the decision-making process of persons who consider themselves unwell is the main focus of its understanding [2], bringing the study of traditional medicine (TM) to the fore.

TM is defined as the sum total of knowledge, skills, and practices based on the theories, beliefs, and experiences indigenous to different cultures that are used to maintain health, as well as prevent, diagnose, improve, or treat physical and mental illnesses [3]. With its holistic nature consisting medication (herbal) and nonmedication (religious or spiritual) therapies [4], TM has maintained longstanding worldwide popularity and is used by large section of the population in both developed and developing countries. In many developed countries 70% to 80% of the population used some form of Complementary and Alternative Medicine (CAM), and 80% of some Asian and African countries and up to 80% of African population depended on TM to meet their primary health care needs [3]. In East Africa too, TM is the dominant and popular system of health care used by about 80% of the population [5].

The study of illness behavior has many applications in research, clinical care, public health, and social policy [1]. By the same token, in-depth knowledge of illness behavior vis-à-vis TM facilitates the understanding of who uses which services and why they access these services. Ultimately, such understanding assists in disease prevention and treatment as well as health care utility and efficacy through creation of effective health promotion policies and programs. Moreover, research from the concept of illness behavior helps immensely in clarifying critical community-based approaches to public health practice. The current international health policy in relation to CAM, involving a shift in the cultural norms considered desirable to foster, is also geared towards enhancing respect for existing cultural identities and harmony with nature.

Ethiopian medical traditions have been studied, among others, by scholars from various disciplines, history, social anthropology, botany, and medicine [6]. However, almost all of the literatures and statistical figures reviewed revealed that the information available dealt with the crude, both preventive and curative, usage of TM. Besides, most of them were concerned with an aspect of TM, herbal medications, studied from biomedical perspectives. Some of these were ethnobotanical studies emphasizing medicinal plants' genera and family distribution, preparation method and administration mode, while others assessed the history, current status, role, major characteristics, and practices [5, 7–11]. Studies also documented medicinal plants and spiritual remedies [12] and assessed patterns of TM utilization [13] in Gondar but confined their study populations to Orthodox Christians and HIV/AIDS patients, respectively.

With regard to predictors of TM practice, some literatures theoretically linked the affordability of TM to the fact of its use by up to 80% of the population [14], while others added poor access to formal health care facilities [15]. Cultural acceptability [7, 16] and local communities' knowledge about TM and its healing procedures [5] have also been mentioned as determinants. Yet, there is still dearth of information about the causal link between TM practice and sociodemographic, socioeconomic, sociocultural (such as attitude and normative beliefs), psychosocial, or individual difference and environmental factors. Albeit the existence of studies on Ethiopian TM, it was hardly possible to find an empirical research that associated traditional herbal and religious practices with the concept of illness behavior too. By using the Integrated Model of Behavioral Prediction (IMBP) as a conceptual framework, therefore, this study aimed to assess TM beliefs and practices and to investigate its predictors in illness behavior in Gondar city, northwestern Ethiopia. Such an analysis not only provides information concerning factors influencing a person's decision to perform or not perform the behavior in question but also identifies attitudes, beliefs, and practices that need to be promoted or changed to produce better health outcomes [17].

## 2. Methods

### 2.1. Study Area and Population

A cross-sectional quantitative survey was conducted in Gondar, a city located 727 km northwest of Ethiopia's capital Addis Ababa at an altitude of 2,220 m above sea level with a mean annual rainfall of 1,172 mm. The mean annual maximum and minimum temperature range from 22°C to 30.7°C and 12.3°C to 17.7°C, respectively. Population size of the city was 206,987 in 2007 [18]. People aged 20 years and above constituted the study population.

### 2.2. Sample Size and Sampling Technique

Sample size was computed using the statistical formula to estimate a single population proportion [19] by assuming 80% (the general prevalence of TM usage in Ethiopia) of the people used TM to deal with their illness experiences. The probability of obtaining this proportion within 5% margin of error was 95%. Using the statistical package Epi_Info Statcalc utility, the required sample size was 246. As the sampling technique used was Probability Proportional to Size (PPS) cluster sampling technique, design effect of 2 was considered to obtain the sample size required to assess statistical significance. By dividing the obtained sample size (492) by 8 sample “kebeles” (the bottom level administrative unit), 61.5 (≈62) households in each kebele were selected, making the final total sample size 496.

### 2.3. Variables and Measurements

Data were collected on a total of 24 variables. The outcome variable was utilization of TM and 2 other variables (types of both remedial action and TM) were considered for descriptive purpose, while data on illness experience was gathered to determine cases to be included into the analysis model. Taking the IMBP as a benchmark, the rest 20 were predictor variables divided into sociodemographic, socioeconomic, sociocultural, individual difference and environmental factors. Sociodemographic characteristics dealt with sex, age, educational level, marital status, religion, and ethnicity; socioeconomic ones included economic status (absolute and subjective poverty) and treatment costs (of both TM and Modern Medicine (MM)); sociocultural determinants were normative response, efficacy belief, attitude, and cue to action by primary group; psychosocial (individual difference) variables consisted of past experience with TM, perceived efficacy, perceived severity/risk, and traditional understanding of illness causation; and environmental factors denoted availability of TM and accessibility of MM.

### 2.4. Data Collection and Quality Control

An interviewer-administered structured questionnaire prepared in English and translated into Amharic was used to collect primary data. Training on the aim, tool, and procedures was given to 16 data collectors (2 in each of the 8 sample kebeles). Beside pretesting of the questionnaire prior to data collection, data quality was ensured through careful facilitation and close supervision.

### 2.5. Data Processing and Analysis

After the collected questionnaires were checked visually for completeness, they were coded and entered into Statistical Package for the Social Sciences (SPSS) for both descriptive and inferential analyses. Frequency run and double data entry on 10% of the questionnaires were performed to check data entry errors. The description involved use of frequencies (*n*) and percentages (%). Analytical inferences were conducted using bivariate and multivariate binary Logistic Regression (LR). Odds Ratio (OR) along with 95% Confidence Interval (CI) was used to assess strength and statistical significance of associations, respectively.

The LR model was given by ln (*p*/(1 − *p*)) = *α* + *β*_1_*x*_1_ + ⋯+*β*_*q*_*x*_*q*_ (or *p* = *e*^*α*+*β*_1_*x*_1_+⋯+*β*_*q*_*x*_*q*_^/(1 + *e*^*α*+*β*_1_*x*_1_+⋯+*β*_*q*_*x*_*q*_^) in terms of probability). Parameters in the model were estimated by maximum likelihood estimate; competing models were compared by score test, likelihood ratio test, and Wald's test; and validity of the model was checked through Omnibus test of model coefficients, the −2 log likelihood statistics, the Hosmer and Lemeshow test, and validation of predicted probabilities. All signified that the incorporation of predictor variables in the model brought significant change between the null model and the model that included both the explanatory variables and the constant, the model providing an adequate fit to the data, and the adequacy of the model to provide a valid prediction. Results were presented using tables, bar graphs, and pie charts.

### 2.6. Ethical Considerations

Ethical clearance was secured from the ethical review committee of the School of Sociology and Social Work, University of Gondar (UoG). Permission and informed consent were obtained from each sample kebele administrator and study participant, respectively. Before obtaining her/his complete verbal consent to participate in the study, each respondent was thus informed about purpose of the study and confidentiality of all information that would be ensured by using codes instead of any personal identifiers.

## 3. Results

### 3.1. Background Profile of Respondents

#### 3.1.1. Sociodemographic Characteristics

A total of 496 respondents participated in the survey, among whom 262 (52.8%) were males. In terms of age, 53.6% and 32.3% of the respondents were, respectively, within the age groups 20 to 35 and 36 to 50. Data on the educational status of respondents showed that 13.3% were uneducated while 39.1% attained higher education. Regarding their marital status, 47.2% were single and 44.4% were married. Majority of the respondents (75.6%) were Orthodox Christians while 16.1% were Muslims (Table 1).

#### 3.1.2. Socioeconomic Characteristics

**Figure 1:**
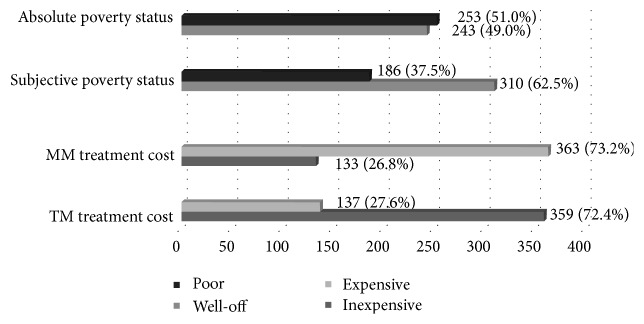
Socioeconomic characteristics of respondents (*n* = 496).

In terms of own interpretation of economic status, subjective poverty, 37.5% perceived themselves as poor. In comparison, the absolute poverty status (i.e., what people need to physically survive) of respondents showed that 51% were poor. Regarding treatment costs, which referred to the monetary cost of getting TM or MM based on respondents' own judgment, majority (72.4%) reported that TM treatment cost was inexpensive, and 73.2% reported that cost of MM was expensive (Figure 1).

#### 3.1.3. Sociocultural Characteristics

**Figure 2:**
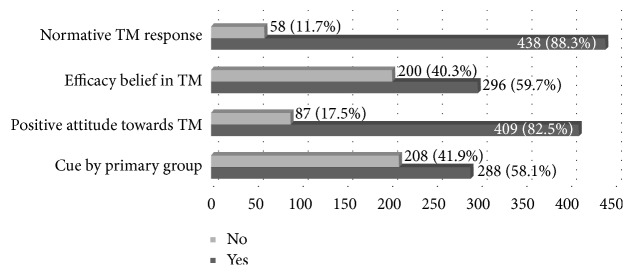
Sociocultural characteristics of respondents (*n* = 496).

Concerning normative response to illness, 88.3% of respondents said that there was a normative traditional way of responding to illness. The results also showed that 59.7% of participants reported that there was an efficacy belief in TM, and attitude towards TM in their locality was positive for 82.5%. Concerning cue to action, 41.9% responded that there was no cue that made them use TM to cure the illness they encountered (Figure 2).

#### 3.1.4. Psychosocial (Individual Difference) Characteristics

Regarding past experience, 79.4% of respondents were satisfied by the treatment they got through TM. Concerning perceived efficacy of TM treatment, 75.6% of respondents perceived using TM to treat illnesses as effective. When their perceived severity or risk of illness encountered in the past one year was taken into consideration, 65.9% of respondents perceived their illness condition as easy. Referring to traditional understanding of illness causation, which was operationalized in this study to refer to the belief that illnesses encountered within the past year were explained in personalistic ways such as spirit possession, loss or damage, soul loss, will of God/Allah or problem with supernatural beings, curse including bewitching, intrusion of foreign objects, and so on, 230 (46.4%) respondents understood the causes of illnesses in traditional manners (Table 2).

#### 3.1.5. Environmental Characteristics

**Figure 3:**
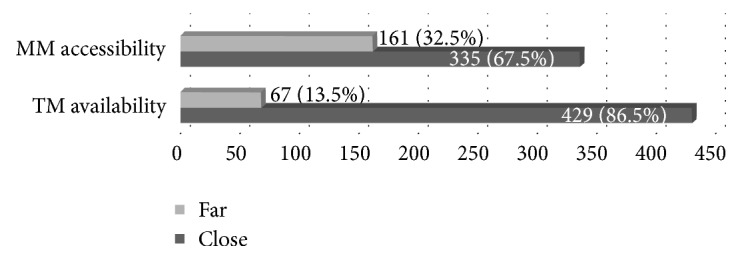
Characteristics of respondents in terms of environmental constraint variables (*n* = 496).

With reference to environmental variables, TM was available for 86.5% of the respondents. The accessibility in terms of physical distance of MM centers was described by 161 (32.5%) respondents as far (Figure 3).

### 3.2. TM Utilization in Illness Behavior

#### 3.2.1. TM Practice and Illness Experience

**Figure 4:**
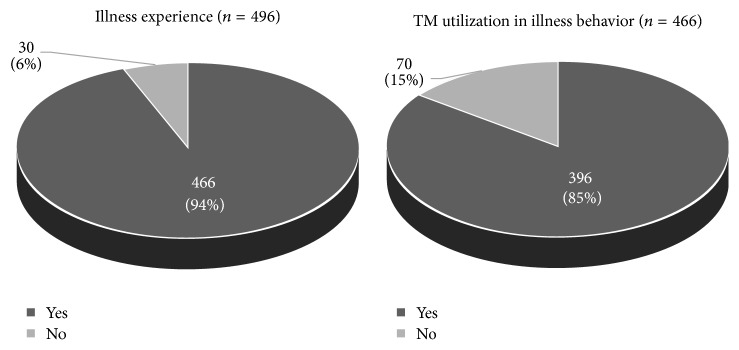
Respondents' illness experience and TM utilization in illness behavior.

Respondents were asked whether they experienced illness of any kind during the past one year. Only 6% reported they did not experience any and thus did not seek medical care. Consequently, they were excluded from further analysis dealing with determinants of TM in illness behavior. The rest 396 (85%) respondents used some form of TM treatments when they felt ill within the past twelve months (Figure 4).

#### 3.2.2. Type of TM and Remedial Action

Among the 466 respondents who experienced illness and sought remedies, 63.7% of them used some sort of traditional herbal medication while 75.3% resorted to religious practices. When the kind of remedial action (through which TM was utilized) was particularly taken into account, the traditional herbal treatments were taken through self-care only (25.8%) and obtained from herbal practitioners only (3.6%). The rest 34.3% used herbal treatments administered by both their own (self-care) and traditional practitioners. The second type of TM, religious healing practice, was performed as self-care (13.7%) and by religious fathers or persons (7.1%). The remaining 54.5% turned to religious healing practices performed both by themselves and spiritual healers (Table 3).

### 3.3. Predictors of TM Practice in Illness Behavior

#### 3.3.1. Unadjusted Binary LR Analysis

A total 466 cases were included in the LR analysis model without any missing cases. Unadjusted binary LR analyses of the outcome variable, utilization of TM in illness behavior, and each potential predictor variables were made. All the sociodemographic variables individually included into the model were statistically insignificant to account for predicted change in TM utilization. Similarly, both subjective and absolute poverty statuses from socioeconomic and perceived severity from psychosocial variables were excluded from inclusion into the adjusted model as their *p* values were beyond the cut-off point.

#### 3.3.2. Adjusted Multivariate LR Analysis

Eleven variables which were significantly associated with TM during the bivariate analysis were entered to the multivariate LR model. TM cost from socioeconomic variables had statistically significant association with TM utilization (*p* = 0.046). The odds of using TM for respondents who reported that its monetary cost was expensive was 0.416 times less than the odds of TM among those who said that it was inexpensive. In contrast, MM cost had no relationship with TM practice (*p* > 0.05). From sociocultural variables, the Adjusted Odds Ratio (AOR) of respondents with normative TM response to illnesses was 7.88 (*p* < 0.001) times greater to use TM. Similarly, attitude towards TM was associated with TM practice (AOR = 5.048; CI = 2.16, 11.8). There was also a positive relationship between cue to action and TM practice (*p* = 0.018). That is, the AOR of TM practice was 2.75 times greater for individuals who were initiated by close friends or family members to use any of TM (Table 4).

Among the psychosocial individual difference predictors, past experience was significantly related to the outcome variable (*p* = 0.007) as the AOR of TM use increased by 3.79 times for respondents with satisfactory past experience of TM practice. Traditional understanding of illness causation similarly had a positive coefficient (1.831), indicating its direct relationship with the response variable (*p* = 0.001). The AOR (*e*^1.831^) showed that personalistic understanding of illness causation increased the probability of TM practice to treat perceived abnormalities by 6.24. Perceived efficacy also demonstrated positive relationship with TM utilization (*p* = 0.004). From environmental factors, TM availability was significantly related to TM utilization at *p* = 0.048. On the other hand, the null hypothesis of no relationship between TM practice and MM accessibility could not be rejected based on the data (*p* = 0.053) (Table 4).

## 4. Discussion

Majority of the population (85%) in Gondar city resorted to TM practices in their illness behavior. This percentage was greater than the report of 70% [7] and the widely claimed overall prevalence of TM practices in Ethiopia (80%) [5, 8, 14, 15] for both preventive and curative reasons. And it was less than other reports that 90% of Ethiopians use TM for primary health care [4]. The finding contradicted the widely claimed argument that the TM utilization percentage was more true in rural than urban areas [8] and was rather in line with others that stated the use of TM among the urban population was also very high [10] and that TM coexisted side by side with the cosmopolitan medicine [16].

Both types of TM, herbal and religious, were practiced by the majority. Similarly, a study undertaken in the same study area but with different study population at UoG hospital reported that spiritual therapy and herbal therapy were the most frequently used TM modalities [13]. Such prevalent utilization of combination of religious and herbal medications could be explained by the facts that Ethiopian traditional life has been painted with the hallmark of widespread use of traditional medicinal plants and blended with religious thinking [7] and almost every aspect of TM remained rooted in and interwoven with religious beliefs and empirical knowledge from the natural environment [16].

The results also indicated that the number of respondents who used traditional herbal medication was outnumbered by those who turned to religious practices. This was in line with a finding that the type of commonly used TM among HIV/AIDS patients was spiritual followed by herbal therapy [13] and was against the assertion that majority of Ethiopians who used TM predominantly relied on medicinal plants [8, 15]. Such differences may be due to domination of one over the other in different cases and areas [16]. And, the outcome that most TM practices were religious may be because of many Ethiopians' belief that their religion helped keep them healthy [12, 14, 20] and Ethiopian TM first and foremost put its emphasis on supernatural forces [6]. A related outcome of the study regarding the kind of remedial action taken in illness behavior was that most TM therapies were utilized through self-care.

Besides disclosing the magnitude of TM utilization in illness behavior, the research also pointed out predictors of such wide practice. Socioeconomic, sociocultural, individual difference and environmental factors proved to be determinants of the type of illness behavior expressed in seeking care, TM practice, and the most important contribution came from sociocultural and individual difference (psychosocial) factors. Other reports similarly stated that culture shaped illness recognition, confidence in the efficacy of treatments, and ultimately illness treatment itself, and TM practice has been strongly related to the rich cultural beliefs of Ethiopia, explaining the emphasis of its use [6, 7, 10].

The normative response pattern that highly favored TM practices was a sociocultural factor that had profound influence in making people use TM to treat their illness experiences. In line with this, literatures explained that illness behavior was found to vary with different learning experiences of conventional coping methods in a given culture [2], be learned through socialization in families and peer groups [21], and arise from learned patterns of response [1]. The traditional understanding of illness causation from individual difference variables was the next important determinant factor. Others also expounded that processes of symptom appraisal were influenced by knowledge [21] and understanding of the cause of the illness was related to TM practices [16]. Such personalistic perceptions have been attributed to the belief that supernatural forces are involved in causing diseases as well as in their treatments [14] and that people believed health is gift of God/Allah and evil forces cause diseases. These interpretations, in light of knowledge, experiences, and other related beliefs, then played a role in the initiation of care, TM practices in this case. On top of these, the positive attitude towards TM significantly added to the prevailing TM practices. According to this study, direction by primary group members as a cue to resort to TM sources was also predictor of the widespread TM utilization.

Moreover, considerable influence on people's decision to utilize TM was presented by psychosocial variables involving differences between individuals, as perceived efficacy of using TM was of an important effect. Congruently, a study reasoned that the trust in the medicinal values of TM was the reason behind its high demand in Ethiopia [7]. Furthermore, respondents' satisfactory past experience with the outcome of TM treatment was positively related to its utilization in illness behavior. This finding supported others that explained illness behavior was influenced by past experiences [21] and TM practices were associated with previous experiences with various health care alternatives [16]. Lastly, an inverse relationship was observed between perceived economic barrier regarding TM utilization and its practice in illness behavior. That is, people turned to TM treatments because they considered the monetary cost required as inexpensive or affordable. Other studies similarly linked the affordability and relatively low cost of using TM with its extensive practice [7, 14]. The monetary cost of taking action was also mentioned as a determinant factor in illness behavior [1].

## 5. Conclusion

Majority of the population in Gondar city relied on both herbal and spiritual varieties of TM to respond to perceived illnesses. The illness behavior of the people can thus be characterized as mainly involving TM practices. The most important predictors of such wide TM practice were sociocultural and psychosocial individual difference variables which, respectively, included normative traditional medical response and positive attitude towards TM and traditional understanding of illness causation, perceived efficacy of TM, and satisfactory past experience with TM. The socioeconomic factor of TM treatment cost and the environmental variable of availability of TM in vicinity were also associated with TM practice.

The results implied two-way medicine needs to be supported and developed with ongoing research, for planning and integrating interventions in the health care system in ways that the general public accepts yielding a better health outcome. Conducting workshops and training by the health bureau and UoG to facilitate collaboration and communication among providers of TM and MM is also deemed necessary. In addition, incorporating TM into the mainstream health education curricula helps meet this goal. Because self-care was the main way of seeking remedy, the health bureau should accord due concern to aware and educate the public about safe usage, particularly of herbal treatments. And, since traditional understanding of illness and normative TM response were predictors of TM practice, health educations must take these customs into consideration. Finally, findings of the study can serve as starting point for future research; while exploring variables of the study is central to understanding predictors of TM practice in illness behavior, other factors such as the influence of social networks in the utilization of or abstinence from TM in response to illness should be investigated. Moreover, since this study was from the perspective of users, further researches from the view point of providers need to be carried out.

## Figures and Tables

**Table 1:** Sociodemographic characteristics of respondents (*n* = 496).

Variable	*n* (%)
Sex	
Male	262 (52.8)
Female	234 (47.2)
Age group	
20–35	266 (53.6)
36–50	160 (32.3)
>50	70 (14.1)
Educational level	
Uneducated	66 (13.3)
Primary education	96 (19.4)
Secondary education	140 (28.2)
Higher education	194 (39.1)
Marital status	
Single	234 (47.2)
Married	220 (44.4)
Divorced	17 (3.4)
Widowed	25 (5.0)
Religion	
Orthodox Christian	375 (75.6)
Muslim	80 (16.1)
Others	41 (8.3)
Ethnicity	
Amhara	414 (83.5)
Tigre	59 (11.9)
Others	23 (4.6)

**Table 2:** Individual difference (psychosocial) characteristics of respondents (*n* = 496).

Variable	*n* (%)
Past experience with TM	
Satisfied	394 (79.4)
Dissatisfied	102 (20.6)
Perceived efficacy	
Effective	375 (75.6)
Ineffective	121 (24.4)
Perceived severity/risk	
Easy	327 (65.9)
Severe	169 (34.1)
Traditional understanding of illness causation	
Yes	230 (46.4)
No	266 (53.6)

**Table 3:** Type of remedial action taken and TM utilized in illness behavior (*n* = 466).

Variable	Traditional medicine utilized	
	Herbal	Religious
	*n* (%)	*n* (%)
Remedial action taken in illness behavior		
Self-care only	120 (25.8)	64 (13.7)
Either herbal or religious practitioners only	17 (3.6)	33 (7.1)
Both self-care and by practitioners	160 (34.3)	254 (54.5)

**Table 4:** Adjusted predictor variables associated with TM practice in illness behavior (*n* = 466).

Variable	*β* coefficient	Wald	*p* value	AOR (95% CI)
Socioeconomic				
TM cost	−.876	3.99	.046	.416 (.176, .984)
MM cost	.832	2.41	.121	2.299 (.803, 6.58)
Sociocultural				
Normative TM response	2.065	15.22	.000	7.88 (2.79, 22.24)
Efficacy belief	−.724	1.71	.191	.485 (.164, 1.434)
Attitude toward TM	1.619	13.92	.000	5.048 (2.16, 11.8)
Cue by primary group	1.012	5.62	.018	2.751 (1.19, 6.35)
Psychosocial				
Past experience with TM	1.333	7.36	.007	3.794 (1.45, 9.95)
Perceived efficacy	1.542	8.26	.004	4.67 (1.63, 13.36)
Traditional understanding of illness causation	1.831	11.3	.001	6.24 (2.15, 18.16)
Environmental				
Availability of TM	1.039	3.91	.048	2.827 (1.01, 7.91)
Accessibility MM	1.390	3.73	.053	4.015 (.98, 16.46)

## Acronyms

AOR: Adjusted Odds Ratio
CAM: Complementary and Alternative Medicine
CI: Confidence Interval
IMBP: Integrated Model of Behavioral Prediction
LR: Logistic Regression
MM: Modern Medicine
OR: Odds Ratio
PPS: Probability Proportional to Size
SPSS: Statistical Package for the Social Sciences
TM: Traditional medicine
UoG: University of Gondar.
